# Characterization of elevated levels of endometrial renin–angiotensin system components suggest a role in endometrial repair

**DOI:** 10.3389/fendo.2026.1817846

**Published:** 2026-04-28

**Authors:** Tess L. Symington, Joshua J. Fisher, Wei Zhou, Evdokia Dimitriadis, Eugenie R. Lumbers, Paul Tooney, Kirsty G. Pringle

**Affiliations:** 1 School of Biomedical Sciences and Pharmacy, College of Health, Medicine and Wellbeing, University of Newcastle, Callaghan, NSW, Australia; 2 Reproductive and Family Health Research Program, Hunter Medical Research Institute, New Lambton Heights, NSW, Australia; 3 School of Medicine and Public Health, College of Health, Medicine and Wellbeing, University of Newcastle, Callaghan, NSW, Australia; 4 Department of Obstetrics and Gynecology, University of Melbourne, Parkville, VIC, Australia; 5 Gynaecology Research Centre, Royal Women’s Hospital, Parkville, VIC, Australia; 6 Mark Hughes Foundation Centre for Brain Cancer Research, Hunter Medical Research Institute, New Lambton Heights, NSW, Australia

**Keywords:** angiotensinogen, endometrial cycle, endometrium, prorenin, prorenin receptor, renin-angiotensin system

## Abstract

**Introduction:**

Each month, the endometrium is shed, regenerated, and transformed in response to changing hormone levels. These processes occur in a tightly regulated manner, and irregularities are linked with reproductive health conditions such as endometriosis and infertility. The renin–angiotensin system is known to be under hormonal control and to drive proliferation and differentiation in tissues, but its expression across the endometrial cycle is not well characterized. Therefore, this study aimed to describe the spatial–temporal localization and gene expression of key initiators of renin–angiotensin system signaling, prorenin, the prorenin receptor, and angiotensinogen, across the endometrial cycle.

**Methods:**

Endometrial tissue was collected from participants within the proliferative phase, mid-secretory phase, and late-secretory phase of their endometrial cycle (n=10/phase). Prorenin (*REN*), the prorenin receptor (*ATP6AP2*), and angiotensinogen (*AGT*) mRNA levels were quantified by qPCR. The spatial localization of proteins was determined using immunohistochemistry and immunolabeling quantified using HALO image analysis software.

**Results:**

Both prorenin (*REN*) mRNA expression and protein immunolabeling intensity were detected at low levels within endometrial tissue, where cell type significantly affected immunolabeling intensity. The mRNA expression of *ATP6AP2* was higher in the proliferative phase compared to the late-secretory phase, with strong prorenin receptor protein immunolabeling across the endometrial cycle, most intensely in the glandular epithelium. *AGT* mRNA expression was higher in the proliferative phase compared to the late-secretory phase endometrium. Mixed effects analysis demonstrated that the angiotensinogen protein labeling intensity was influenced by endometrial cell type but not cycle phase and was highest in the endometrial stroma. There was, however, an interaction between cell type and phase whereby angiotensinogen immunolabeling in the stroma tended to be stronger in the mid-secretory phase compared with the late-secretory phase.

**Conclusion:**

*AGT* and *ATP6AP2* mRNA levels are higher in the proliferative phase compared to the late-secretory phase of the endometrial cycle. Angiotensinogen immunolabeling is highest in the stroma regardless of cycle phase and tends to be lower in the stroma of the late-secretory phase compared to the mid-secretory phase. These findings suggest that the renin–angiotensin system may play a regulatory role in driving stromal regeneration and proliferation in the proliferative and mid-secretory phase of the endometrial cycle.

## 1 Introduction

Each month, the endometrium undergoes cyclic remodeling in response to changes in estrogen and progesterone levels, in order to prepare for implantation and pregnancy (1). The endometrial cycle is on average a 28-day cycle (1) characterized by both functional and morphological changes of the basal and functional layers of the endometrium that are composed of stroma, spiral arteries, immune cells, and uterine epithelial glands and lined by the luminal epithelium.

The endometrial cycle begins with the menstrual phase, in which declining estrogen and progesterone levels induce coiling and constriction of the uterine spiral arterioles, resulting in tissue ischemia and an inflammatory response that causes shedding of the functional layer of the endometrium. Following the menstrual phase, the endometrial cycle enters the proliferative phase, characterized by the regrowth and repair of the functional layer in response to increasing estrogen levels. The secretory phase is then initiated by ovulation, when increasing levels of progesterone drive decidualization throughout the endometrium, to allow for embryo implantation and subsequent placentation. If implantation does not occur, levels of progesterone and estrogen drop, initiating the start of the cyclic remodeling again. The whole endometrial cycle occurs in a tightly regulated manner (1), and dysregulation of the cycle can result in an array of reproductive and gynecological disorders, including infertility and miscarriage, as well as unopposed estrogen levels, which can lead to endometrial cancer (2–4). In order to fully understand what is dysregulated within the endometrium when reproductive health conditions and imbalances in hormones occur, there needs to be a greater understanding of the factors coordinating endometrial remodeling and their temporal regulation.

The renin-angiotensin system is expressed locally within the endometrium and is postulated to play an important role across the endometrial cycle (5–7). Although most commonly recognized as a circulating system known to control blood pressure and salt and water balance, specific tissue renin–angiotensin systems exist, including within the endometrium (8–11). The renin–angiotensin system pathway is initiated when active renin acts on angiotensinogen (AGT) to form angiotensin I (Ang I). Within tissues, the most common form of renin is prorenin, an inactive form in which the active site of renin is covered by the pro-segment. Prorenin can be activated by cold temperature, acidity, proteases, or when prorenin binds the prorenin receptor (PRR), all of which result in the unfolding/removal of the pro-segment (12–14). Once Ang I is generated, it is converted into Ang II via the angiotensin-converting enzyme (ACE). Subsequently, Ang II can act via the angiotensin II type 1 receptor (AGTR1) or angiotensin II type 2 receptor (AGTR2) to promote signaling pathways that stimulate cellular proliferation and angiogenesis, processes important for the cyclic remodeling of the endometrium.

It has been well established that components of the circulating renin–angiotensin system are affected by the levels of both progesterone and estrogen (5, 9, 15–21) and that components including renin, ACE, Ang II, AGTR1, and AGTR2 change across the endometrial cycle (8, 9, 22). Renin has been reported to show constant epithelial immunostaining across the endometrial cycle, with the strongest stromal staining in the proliferative phase and no detectable endothelial staining throughout (8). ACE immunostaining is strong throughout the endometrium across the whole cycle (8), with stromal Ang II, AGTR1, and AGTR2, along with glandular epithelial Ang II being strongest in the proliferative phase (9). Both AGTR1 and AGTR2 show consistent glandular epithelium and endothelium expression (9, 22). However, the changes to prorenin, the most common form of renin in the endometrium, the prorenin receptor (PRR), which has been shown to be dysregulated in hormonal disorders, including endometrial cancer (23), and AGT, which is known to be under the influence of estrogen (16, 17), have not been characterized within the healthy, cycling endometrium.

This study aimed to determine the changes that occur to the mRNA and protein levels of prorenin, its receptor, PRR, and its substrate, AGT, across the endometrial cycle. Endometrial tissues were collected from patients within the proliferative phase, mid-secretory phase, and late-secretory phase of their endometrial cycle. *REN* (prorenin), *ATP6AP2* (PRR), and *AGT* mRNA expression was measured by qPCR and prorenin, and PRR and AGT protein localization and immunostaining intensity were determined using immunohistochemistry.

## 2 Materials and methods

### 2.1 Ethics

This study was approved by the Human Research Ethics Committees at the Hudson Institute and at the Royal Women’s Hospital (ID: #03066B). All participants provided written informed consent, were aged between 20 and 50 yrs old, had regular endometrial cycles (21–35-day cycles), had no indications of infertility, were not using intrauterine contraceptives, had no hormonal treatments for at least 3 months prior to surgery, and histopathology confirmed the absence of endometrial dysfunction such as endometriosis. Collected endometrial samples were assessed by gynecological pathologists to determine the cycle stage based on standard histological criteria (24).

### 2.2 Tissue collection

Endometrial tissues were collected from participants undergoing endometrial curettage and snap frozen or 10% formalin fixed within 1 h of surgery. Samples were then grouped into the proliferative, mid-secretory, and late-secretory phases of the endometrial cycle (n=10/phase) based on cycle staging by gynecological pathologists.

### 2.3 Real-time reverse transcriptase polymerase chain reaction

Total RNA was isolated using an RNeasy Mini Kit (Qiagen, Germantown, MD, USA) for endometrial tissues following the manufacturers’ instructions. DNA contamination was removed using the TURBO DNase Kit (Thermo Fisher Scientific, Waltham, MA, USA) according to the manufacturer’s protocol. Total RNA was then quantified using a NanoDrop One spectrophotometer (Thermo Fisher Scientific). A total of 1 μg of RNA per sample was reverse transcribed using a SuperScript IV RT kit with random hexamers (Invitrogen, Thermo Fisher Scientific). qPCR was performed using an Applied Biosystems QuantStudio 6 Flex Real-Time PCR System (Thermo Fisher Scientific) to detect target genes using SYBR Green (Thermo Fisher Scientific). Samples were run in duplicate, with genes measured described in **Supplementary Table 1**. Target gene mRNA expression was calculated using the 2^−ΔΔCT^ method relative to the geometric mean of three housekeeping genes: β-actin (*ACTB*), Tyrosine 3-Monooxygenase/Tryptophan 5-Monooxygenase Activation Protein Zeta (YWHAZ), and 18S rRNA (*18SrRNA*), with a reference sample run across all plates (term human decidua).

### 2.4 Immunohistochemistry

Formalin-fixed tissues were paraffin-embedded and cut into four-micron-thick sections. Tissue slides then underwent dewaxing using xylene and ethanol. Endometrial tissue sections then underwent antigen retrieval in 10 mM citrate buffer at pH 6.0 at 90°C for either 10 min (PRR) or 30 min (renin pro-peptide (prorenin) and AGT). Following antigen retrieval, endometrial tissue sections were cooled before being washed in PBS three times for 5 min per wash. Endometrial tissue samples then underwent two 30min rocking endogenous peroxidase blocks (3% hydrogen peroxide in 0.1 M PBS) at room temperature. Tissue sections were then washed three times in PBS before being incubated in blocking solution (0.5% bovine serum albumin (BSA) in 0.1 M PBS) for 1 h at room temperature. Following incubation with blocking solution, endometrial tissue sections were incubated overnight at 4°C with the primary antibodies (renin pro-peptide 2.5μg/mL, R&D Systems, Minneapolis, MN, USA, MAB4447, RRID: AB_2238435; PRR 0.2μg/mL, Everest Biotech, Oxfordshire, UK, eb06118, RRID: AB_2062088; AGT 0.02μg/mL, R&D Systems, af3156, RRID: AB_2225450). Endometrial tissue sections that were used as negative controls were matched samples that did not undergo primary antibody incubation and were instead incubated in blocking solution. After overnight incubation, endometrial tissue sections were washed three times with PBS and then incubated with a secondary antibody. PRR and AGT samples were incubated with a rabbit anti-goat antibody (Sigma-Aldrich, St. Louis, MO, USA, B7024 diluted to 1:300; RRID: AB_258599), and renin propeptide samples were incubated with a rabbit anti-mouse antibody (Abcam, Cambridge, UK; 1mg/mL diluted 1:300; ab97044, RRID: AB_10687624) for 1 h at room temperature. Following secondary antibody incubation, endometrial sections were washed in PBS three times and then incubated with the streptavidin–biotin–horseradish peroxidase complex (Abcam, diluted 1:400 in 0.1 M PBS; ab7403) for 1 h at room temperature. Endometrial tissue sections were washed three times in PBS before being stained with 3-3'-diaminobenzidine tetrahydrochloride solution (Pierce, Thermo Fisher Scientific; metal enhanced DAB substrate kit #34,065) with 0.01% hydrogen peroxide in 0.1M PBS for 10 min for PRR and AGT, and 20 min for renin propeptide (Santa Cruz, California, USA; sc-209686B). The endometrial sections were then washed three times in PBS and counterstained with Hematoxylin (Merck-Millipore, Burlington, MA, USA; Gill No. 2; GHS216), for 30 sec followed by three dH_2_O washes. Endometrial sections were then dehydrated in ethanol and xylene before being mounted using DEPX (Merck-Millipore). Each microscope slide was imaged using the Aperio AT Turbo slide scanner (Leica Biosystems).

### 2.5 Image analysis

Immunohistochemistry images were analyzed using HALO software (Indica Labs, Albuquerque, NM, USA). Using HALO, images were segmented by hand to separate stroma, glandular epithelium, endothelial cells, and luminal epithelium, with the staining of blood within the blood vessels excluded from the analysis area (**Supplementary Figure 1**). Stromal cells and glandular epithelium were identified in all 10 patients from each phase. Luminal epithelial cells were observed in 8 of 10 samples from patients within the proliferative phase, and 9 of 10 patient samples in both the mid- and late-secretory phases. Endothelial cells were present in 8 of 10 tissue sections from patients within the proliferative phase, and all 10 patient samples within both the mid- and late-secretory phases. Once segmented, sections were analyzed using HALO software, which can deconvolve images into a hematoxylin channel and a DAB channel and generated the percentages of the tissue that immunolabelled weak, moderate, and strong. Percentages were then used to calculate an H-score for each cell type, using the following formula as described elsewhere (25);


H-score (1 × weak stain %) + (2 × moderate strain %) + (3 × strong stain %).

The same method was used for secondary antibody only control samples, with the average H-score of up to four secondary antibody-only negative control samples across the three phases subtracted from the H-score of all sections that were immunolabelled with primary antibodies. The H-score generates values between 0 and 300, with scores between 0 and 99 deemed low staining, 100–199 moderate staining, and 200–300 strong staining.

### 2.6 Statistical analysis

Statistical analysis was performed using GraphPad Prism (Version 10). A Shapiro–Wilk normality test was used to determine normality. A ROUT’s outlier test was used to remove outliers before further analysis. For mRNA data, if the average CT values for sample replicates were 32 cycles or above, samples were deemed to have no mRNA expression and were allocated a zero value. For immunohistochemical analysis, any negative H-scores were deemed to have no protein expression and were assigned an H-score of zero. For mRNA expression across the endometrial cycle, parametric data (*ATP6AP2*) were analyzed using a Brown–Forsythe and Welch ANOVA, and non-parametric data (*REN* and *AGT*) were analyzed using a Kruskal–Wallis test, both with multiple comparisons. The effect of cell type and menstrual cycle phase on staining intensity (H-score) was assessed using a mixed-effects analysis with Tukey’s multiple comparisons. Results were deemed significant if p<0.05.

## 3 Results

### 3.1 mRNA changes to the renin–angiotensin system across the proliferative and secretory phases

**Figure 1:**
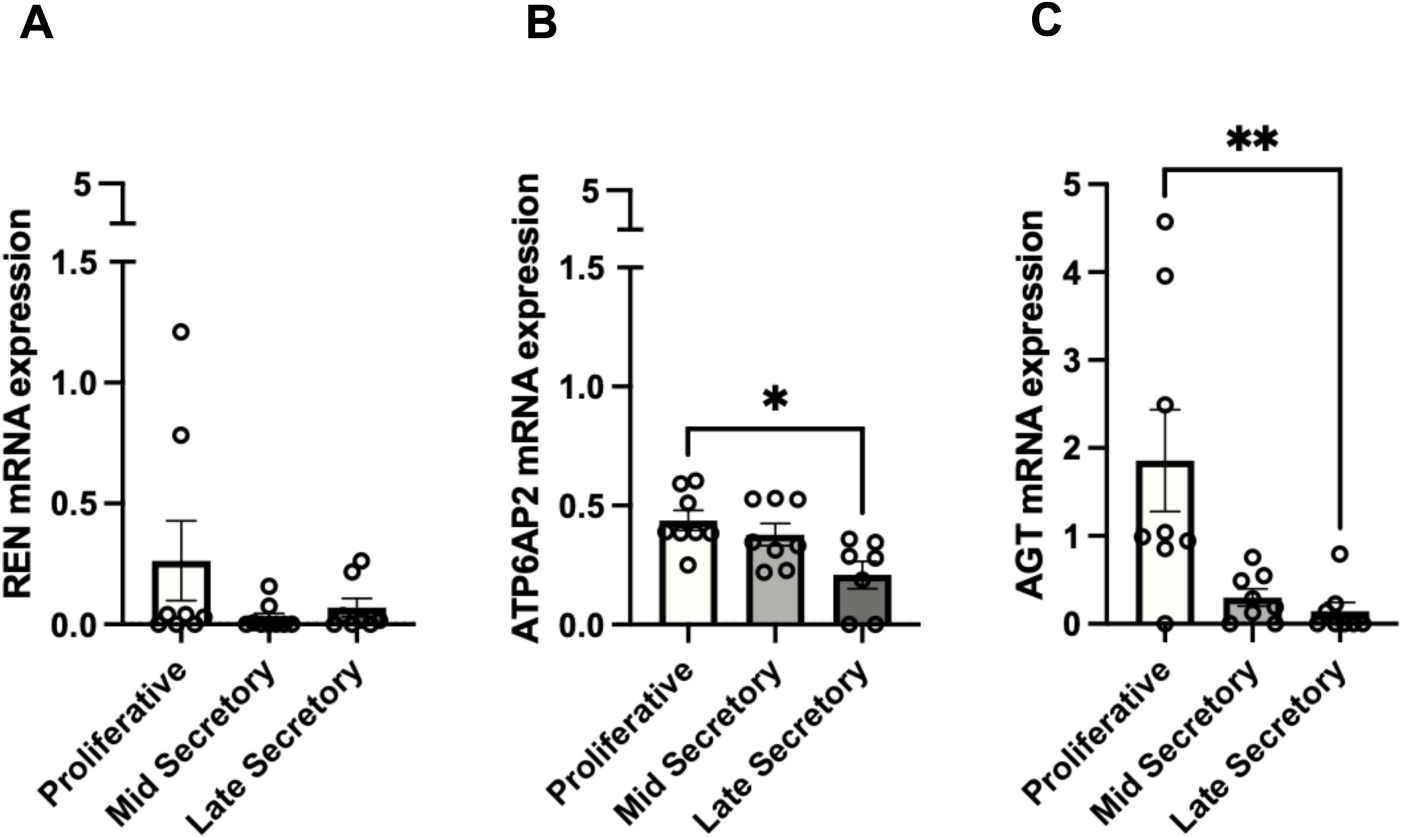
mRNA expression of endometrial renin, ATP6AP2, and AGT within the proliferative and secretory phases of the endometrial cycle. **(A)** mRNA expression of *REN* did not change across the proliferative and secretory phases of the endometrial cycle. **(B)** *ATP6AP2* mRNA expression was significantly higher in the proliferative phase compared to the late-secretory phase (p=0.024). **(C)** *AGT* mRNA expression was significantly higher in the proliferative phase compared to the late-secretory phase endometrium (p=0.004). *AGT* mRNA tended to be higher in the proliferative phase compared to mid-secretory phase endometrium; however, this was not statistically significant (p=0.061). Data are presented as mean ± SEM. mRNA expression was measured in endometrial tissue collected from patients in the proliferative phase (n=8), mid-secretory phase (n=9), and late-secretory (n=8) phases. Data were analyzed using a Brown–Forsythe and Welch ANOVA for parametric data (*ATP6AP2*) and a Kruskal–Wallis test for non-parametric data (*REN, AGT*), all with multiple comparisons. *p < 0.05, **p < 0.01.

To determine the mRNA expression of endometrial *REN, ATP6AP2*, and *AGT* during the proliferative and secretory phases of the endometrial cycle, the levels of these components were assessed by qPCR. Overall, there were no significant differences in *REN* mRNA expression between the three phases of the endometrial cycle (**Figure 1A**). *ATP6AP2* mRNA levels in the endometrium were significantly higher in the proliferative phase compared to the late-secretory phase (**Figure 1B**, p=0.024). The expression of *AGT* mRNA was significantly higher in the proliferative phase compared to the late-secretory phase (**Figure 1C**; p=0.004) and tended to be higher in the proliferative phase compared to the mid-secretory phase; however, this was not statistically significant (p=0.061).

### 3.2 Prorenin protein abundance does not change across the proliferative and secretory phases

**Figure 2:**
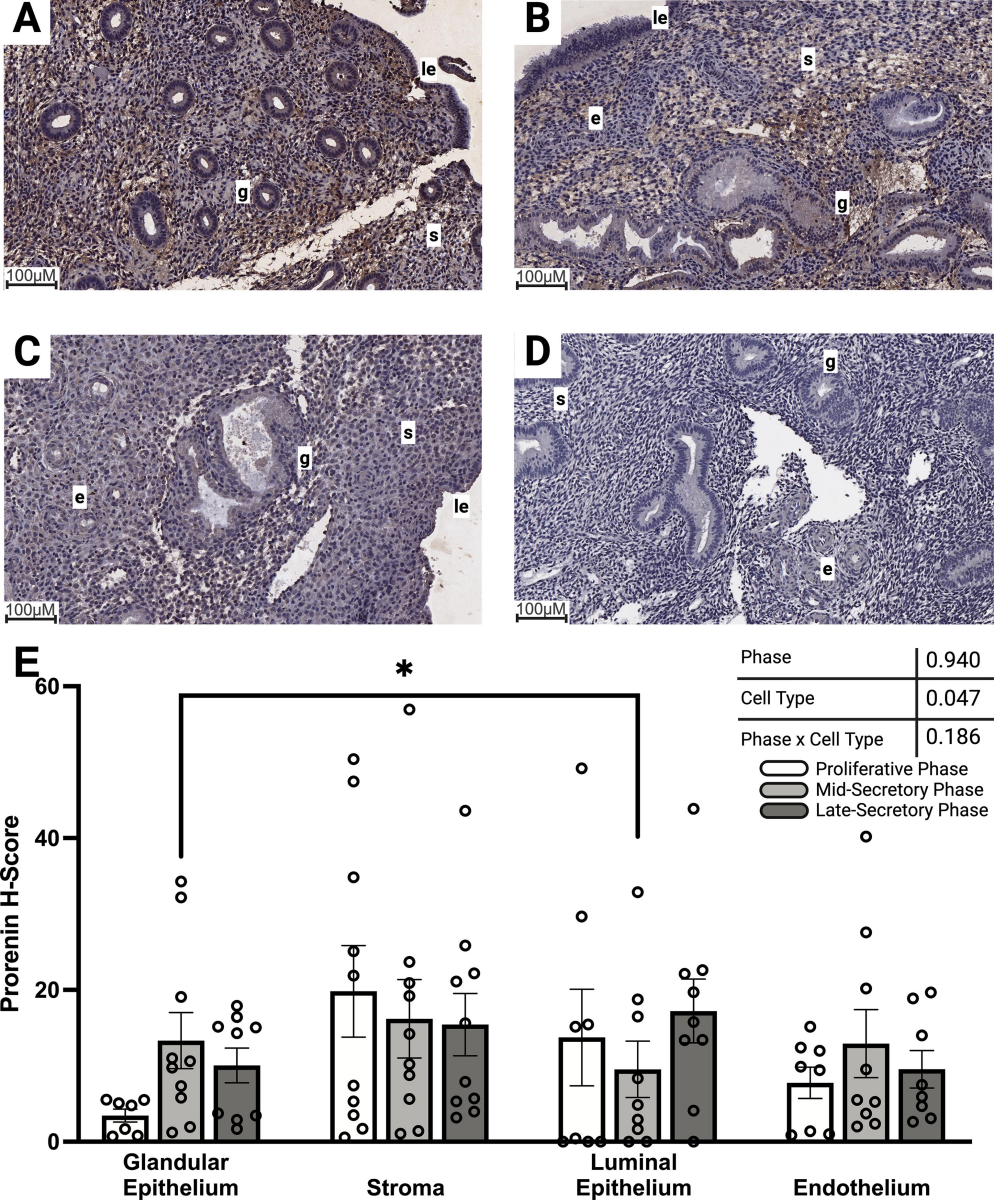
Abundance of prorenin protein within the endometrium during the proliferative and secretory phases. Representative images of prorenin immunolabeling within the **(A)** proliferative, **(B)** mid-secretory, and **(C)** late-secretory phase endometrium. **(D)** Representative image of no primary antibody control-labeled endometrium. All images were captured at 15× magnification. Immunolabeling intensity for prorenin was compared between proliferative phase (n=10 glandular and stroma, n=8 endothelial, and n=8 luminal epithelium), mid-secretory phase (n=10 glandular, stroma, and endothelial, and n=9 luminal epithelia), and late-secretory phase (n=10 glandular, stroma, and endothelial, and n=9 luminal epithelia) endometrial tissue **(E)**. Mixed-effects analysis (ANOVA) showed a significant effect of cell type (p=0.047) and no effect of phase (p=0.940) or an interaction between cell type and phase (p=0.186) on endometrial prorenin labeling intensity. Immunolabeling of prorenin within the mid-secretory phase was more intense in the glandular epithelium than in the luminal epithelium (p=0.028). Brown indicates DAB protein staining; blue indicates hematoxylin nuclear counterstaining. Data were analyzed using mixed-effects analysis with Tukey’s multiple comparisons *p < 0.05, g—glandular epithelium, s—stroma, e—endothelium, le—luminal epithelium. White bars represent the proliferative phase, light gray the mid-secretory phase, and dark gray the late-secretory phase.

Immunolabeling of the prorenin pro-peptide was low, with some areas within the whole tissue showing immunolabeling and others having negligible labeling compared with the negative control (**Figures 2A–D**). There were no significant differences in labeling intensity between the proliferative and secretory phases (**Figure 2E**). Mixed-effects analysis showed that cell type significantly affected prorenin protein immunolabeling (**Figure 2E**, p=0.047). The labeling intensity of prorenin within the glandular epithelium of the proliferative phase endometrium (**Figure 2A**) tended to be weaker than in both the mid-secretory (**Figure 2B**) and late-secretory phases (**Figure 2C**); however, this did not reach statistical significance (p=0.062 and p=0.052, respectively). Within the mid-secretory phase, prorenin immunolabeling intensity was significantly higher in the glandular epithelium than in the luminal epithelium (**Figure 2E**, p=0.028).

### 3.3 Prorenin receptor protein is abundant across the endometrial cycle

**Figure 3:**
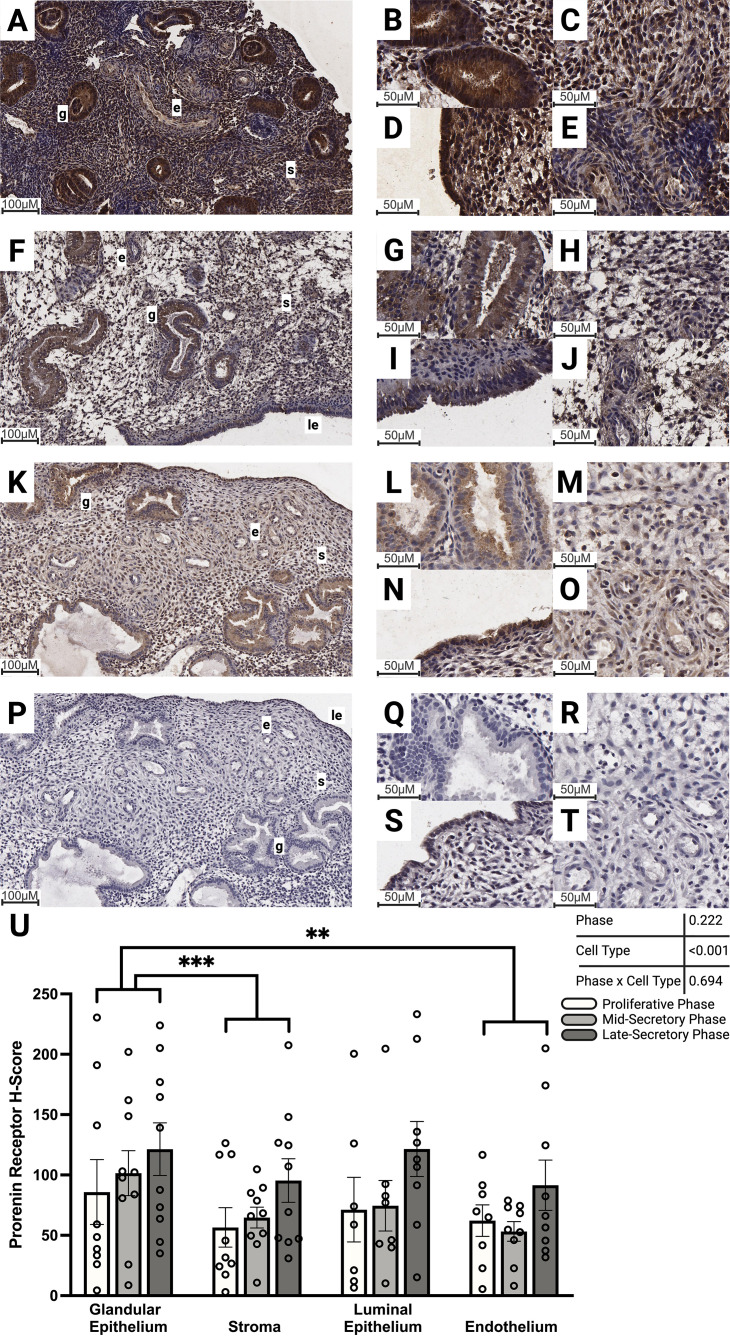
Abundance of PRR protein within the endometrium during the proliferative and secretory phases. Representative images of PRR protein immunolabeling in **(A-E)** proliferative, **(F-J)** mid-secretory, and **(K-O)** late-secretory phase endometrium. **(P-T)** Representative images of no primary antibody control-labeled endometrium. Images **(A, F, K**, **P)** were captured at 15× magnification; close-up images of glandular epithelium **(B, G, L, Q)**, stroma **(C, H, M, R)**, luminal epithelium **(D, I, N, S)**, and endothelium **(E, J, O, T)** were captured at 40× magnification. **(U)** Immunolabeling intensity (mean ± SEM) was compared between proliferative phase (n=9 glandular epithelium and stroma, n=8 endothelium, and n=7 luminal epithelium), mid-secretory phase (n=10 glandular epithelium, stroma and endothelium, and n=8 luminal epithelium), and late-secretory phase (n=10 glandular epithelium and stroma, and n=9 luminal epithelium and endothelium) endometrial tissue. A significant effect of cell type (p<0.001) on PRR immunolabeling intensity was identified by mixed-effects analysis (ANOVA), with no effect of phase (p=0.222) and no interaction found between phase and cell type (p=0.694). Brown indicates DAB protein staining, and blue indicates hematoxylin nuclear counterstaining. Stars denote differences between cell types in the mixed-effects analysis with Tukey’s multiple comparisons **p < 0.01, ***p<0.001, g—glandular epithelium, s—stroma, e—endothelium, le—luminal epithelium. White bars represent the proliferative phase, light gray the mid-secretory phase, and dark gray the late-secretory phase.

Prorenin receptor immunolabeling ranged from weak to moderately present across the proliferative and secretory phases, with homogenous and consistent labeling within patient samples, but variable staining levels between patients (**Figures 3A–U**). Mixed-effects analysis of PRR immunolabeling intensity in the endometrium revealed a significant effect of cell type (**Figure 3U**; p<0.001), but no effect of cycle phase. The glandular epithelium (**Figures 3B, G, L**) of the endometrium had significantly stronger immunolabeling irrespective of cycle phase, compared to both the stroma (**Figures 3C, H, M**; p<0.001) and endothelium (**Figures 3E, J, O**; p=0.006).

### 3.4 Angiotensinogen protein abundance is highest in the proliferative phase endometrium

**Figure 4:**
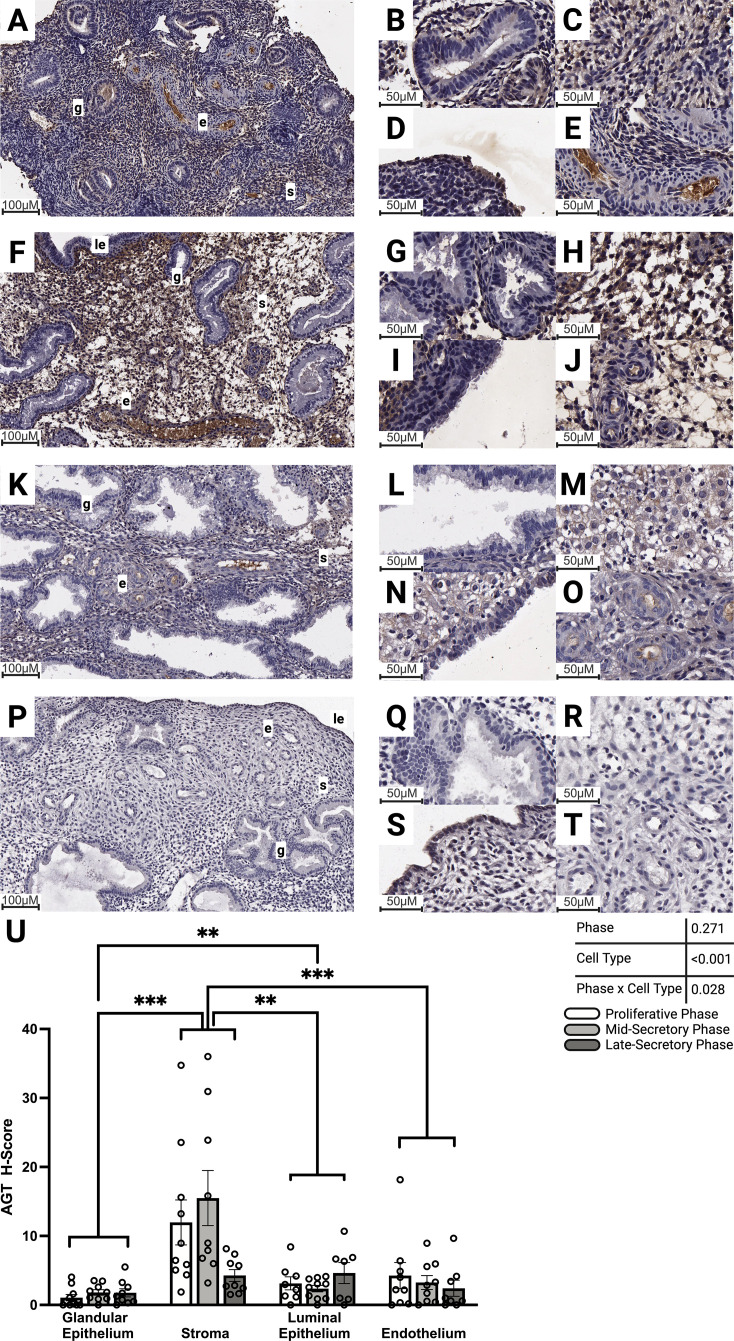
Abundance of AGT protein within the endometrium during the proliferative, and secretory phases. Representative images of AGT protein immunolabeling in **(A-E)** proliferative, **(F-J)** mid-secretory, and **(K-O)** late-secretory phase endometrium. **(P-T)** Representative images of no primary antibody control-labeled endometrium. Images **(A, F, K**, **P)** were captured at 15× magnification, close-up images of glandular epithelium **(B, G, L, Q)**, stroma **(C, H, M, R)**, luminal epithelium **(D, I, N, S)**, and endothelium **(E, J, O, T)** were captured at 40× magnification. **(U)** Labeling intensity (mean ± SEM) was compared between proliferative phase (n=10 glandular epithelium and stroma, n=9 endothelium, and n=8 luminal epithelium), mid-secretory phase (n=10 glandular epithelium, stroma, endothelium, and luminal epithelium), and late-secretory phase (n=10 glandular epithelium and stroma, n=9 endothelium, and n=8 luminal epithelium) endometrial tissue. A significant effect of cell type (p<0.0001) and a significant interaction between cell type and phase (p=0.027) were identified by mixed-effects analysis (ANOVA). Brown indicates DAB protein staining, and blue indicates hematoxylin nuclear counterstaining. Stars denote differences between cell types in the mixed-effects analysis with Tukey’s multiple comparisons **p < 0.01, ***p<0.001. g—glandular epithelium, s—stroma, e—endothelium, le—luminal epithelium. White bars represent the proliferative phase, light gray the mid-secretory phase, and dark gray the late-secretory phase.

Endometrial immunolabeling for AGT was low across the endometrial cycle (**Figures 4A–U**). Mixed-effects analysis revealed that cell type but not cycle phase had a significant effect on endometrial AGT labeling intensity (p<0.001 and p=0.271) and that there was a significant interaction between cell type and phase (**Figure 4U**; p=0.027). Irrespective of cycle phase, immunolabeling of AGT was stronger in the stroma (**Figures 4C, H, M**) than in the glandular epithelium (**Figures 4B, G, L**), luminal epithelium (**Figures 4D, I, N**), and endothelium (**Figures 4E, J, O**; p<0.001, p=0.007, and p<0.001, respectively). AGT immunolabeling in the luminal epithelium was stronger than that of the glandular epithelium (**Figure 4U**; p=0.006). AGT immunolabeling also tended to be stronger in the stroma of the mid-secretory phase endometrium compared to the late-secretory phase; however, this failed to reach statistical significance (p=0.055).

## 4 Discussion

This study aimed to characterize the changes to prorenin (*REN*), PRR (*ATP6AP2*), and AGT mRNA expression and immunolabeling across the proliferative and secretory phases of the endometrial cycle. We showed that both *REN* mRNA and prorenin immunolabeling were present in the endometrium but at low levels across the proliferative and secretory phases. *ATP6AP2* mRNA expression was higher in the proliferative compared to the late-secretory phase endometrium, with low to moderate protein immunolabeling throughout the endometrium, which was found to be significantly higher in the glandular epithelium compared to the stroma and endothelium. *AGT* mRNA expression was highest in the proliferative phase. The intensity of AGT protein immunolabeling was affected by cell type and was strongest within the endometrial stroma.

*REN* mRNA expression and prorenin protein immunolabeling within the endometrium were shown to be low across the proliferative and secretory phases of the endometrial cycle. To the best of our knowledge, this is the first study to examine prorenin (*REN*) mRNA expression and prorenin protein immunolabeling within the endometrium, determining cellular protein localization and abundance. One previous study by Li et al. examined renin, the active form of prorenin, across the endometrial cycle via immunohistochemistry, using a custom renin antibody (8). Strong immunolabeling of renin was demonstrated within the epithelial glands of the endometrium, with moderate stromal immunolabeling in the proliferative phase but negligible immunolabeling across the rest of the cycle, and no endothelial labeling throughout (8). Older studies by Johnson examined both renin and acid-activated renin (prorenin) by radioimmunoassay, across the endometrial cycle, in the plasma and endometrial tissue of both naturally cycling patients and those taking an estrogen-containing oral contraceptive pill (21, 26). Their findings supported the local production of renin and prorenin within the endometrium, with tissue levels varying independently of plasma, and at levels up to 200 times higher (21, 26). In patients taking the estrogen-containing contraceptive pill, levels of renin within the endometrium were increased across the cycle (26), and naturally cycling patients had significantly higher levels of renin in the proliferative phase compared to the secretory phase endometrium (21, 26), supporting estrogen regulation of renin within the endometrium. This is reflected in Li et al.’s findings of higher stromal immunolabeling of renin within the proliferative phase (21), where estrogen is the dominant sex hormone. Johnson then went on to show no significant changes to prorenin across the endometrial cycle (21), which is in support of the findings of this study where we identified no significant differences to both mRNA and prorenin protein immunolabeling across the endometrial cycle. Together, these findings suggest that active renin levels change across the endometrial cycle and are regulated by estrogen, while prorenin levels remain stable.

Although we did not identify any changes to prorenin across the endometrial cycle, we did observe changes to both its receptor, the PRR (*ATP6AP2*), and AGT, where mRNA levels were higher within the proliferative phase compared to the late-secretory phase. It has previously been shown that Ang II immunoreactivity is higher within proliferative phase stroma and glandular epithelium than in the secretory phase (9) and that ACE levels within the endometrium are strongly present throughout (8). This suggests that although prorenin levels are low and remain stable, the stronger mRNA and immunolabeling levels of PRR and AGT could mean a greater capacity for activated prorenin to generate Ang I, as do high levels of renin immunolabeling with consistent ACE and higher Ang II immunoreactivity, suggesting increased RAS signaling within the proliferative phase endometrium.

The low-moderate protein staining for the PRR across the endometrial cycle suggests that it plays an important role in controlling endometrial function, especially within the glandular epithelium, where immunolabeling was found to be the most intense. The PRR is also involved in regulating pathways unrelated to the renin–angiotensin system, including Wnt signaling, V-ATPase actions, and tyrosine phosphorylation, which are known to have roles in proliferation, angiogenesis, fibrosis, migration, and invasion (27, 28), all processes the endometrium undergoes across the endometrial cycle. Indeed, we have previously shown that silencing of the PRR in grade I endometrial cancer cells *in vitro* with an siRNA decreased cellular proliferation and viability (29). To our knowledge, the current study is the first to investigate the PRR within the healthy endometrium. There is little evidence that the PRR is regulated by sex hormones, other than in environments of high estrogen, where the PRR is increased, for example in the plasma of obese women with type 2 diabetes mellitus compared to men (30) and in endometrial cancer, a cancer of the glandular epithelium (23). Furthermore, treatment of endometrial cells with estrogen, progesterone, and cAMP, to induce decidualization, was shown to have no effect on PRR mRNA expression (5). The sustained expression of PRR within the glandular epithelium highlights the key role of PRR in regulating endometrial proliferation and supports the importance of the PRR across the whole endometrial cycle.

AGT is the initial source of all angiotensin peptides. We showed that *AGT* mRNA levels were higher within the proliferative phase compared with the late-secretory phase and tended to be higher compared with the mid-secretory phase. Lower mRNA expression of *AGT* within the late-secretory phase was reflected in the low stromal protein immunolabeling for AGT at this stage. This finding is unsurprising as it is well established that hepatic production of AGT is regulated by estrogen (16, 17, 21, 26), and estrogen is the dominant sex hormone within the proliferative phase with levels decreasing in the secretory phase. In support of our study, Johnson also showed a decrease in AGT levels as the endometrial cycle progressed (21). Together, these data support the role of AGT in helping with the regrowth of endometrial tissue during the proliferative phase, especially in the stroma where the majority of AGT protein immunolabeling was found within the endometrium.

AGT is not the only renin–angiotensin system component known to be regulated by estrogen. Alongside AGT, estrogen has been shown to alter the AGTR1 in numerous tissues, including the endometrium (9, 18–20). Both AGTR1 mRNA and protein have been shown to be highest in the proliferative phase stroma, with consistent epithelial gland and endothelium expression (9, 22). AGTR2 is the most predominant renin–angiotensin system receptor in the endometrium. Ahmed et aal. have demonstrated that greater than 60% of Ang II-specific binding within the endometrium was to AGTR2, and about 20% was to AGTR1 (9). However, both AGTR1 and AGTR2 showed the same patterns of binding for Ang II across the endometrial cycle, with levels increasing across the proliferative phase to be strongest early in the secretory phase before decreasing prior to menstruation (9). Both receptors are acted on by Ang II, which has been shown to be important for endometrial regeneration within the rat endometrium, where blocking of Ang II generation using an ACE inhibitor caused a reduction in endometrial proliferation (7). Subsequent treatment with Ang II and Ang IV increased proliferation within the endometrium (7). Furthermore, there is some evidence that the expression of AGTR1 is dysregulated in endometrial conditions such as endometrial hyperplasia (31, 32), and repetitive implantation failure (33), where receptor levels were shown to be lower. This is in contrast to conditions characterized by excessive growth such as endometrial cancer where AGTR1 was found to be increased (23). Together, these data highlight the role for an optimally functioning endometrial RAS in the regulation of endometrial function.

This study has several limitations, including the mRNA results being limited due to the use of tissue homogenates, which contain all cell types of the endometrium combined. This allowed us to assess the overall changes to renin–angiotensin system mRNA expression across the endometrial cycle within the endometrium, but not within individual cell types. Further studies should utilize single-cell sequencing databases such as the Human Endometrial Cell Atlas (34) to determine the changes to the expression of components of the renin–angiotensin system on a cellular level, across the endometrial cycle. In terms of protein immunolabeling, we were able to segment different cellular compartments within the endometrium, allowing us to do a comprehensive analysis of whole tissue sections, and to generate an H-score to quantify expression within each cell type of the endometrium. Previous protein analysis of renin–angiotensin system abundance has mostly used a four-point scale to characterize staining intensity and failed to include the luminal epithelium (8, 9). Using the H-score allowed us to assess protein abundance in a more robust way, and to statistically quantify the changes across the whole tissue; however, as highlighted throughout, changes within the endometrium are gradual and whole tissue analysis may mask some of the subtle non-uniform changes to protein abundance within pockets of the endometrium. Furthermore, this manuscript focused solely on descriptive analysis and did not assess whether the changes in renin–angiotensin system components are a cause or effect of endometrial proliferation. Therefore, further mechanistic studies are needed to elucidate the direct role of prorenin, the prorenin receptor, and angiotensinogen within the endometrium.

In conclusion, we have shown for the first time that prorenin and AGT proteins are present within the endometrium in low levels; PRR protein is present in high levels; prorenin, PRR, and AGT proteins were affected by cell type; and that *ATP6AP2* and *AGT* mRNA levels were highest in the proliferative phase. Immunolabeling of the PRR was shown to be affected by cell type, but not the endometrial cycle phase, and to be highest in the glandular epithelium, revealing insights into the importance of the PRR within the endometrium and enhancing our understanding of how PRR expression patterns may be altered in disease states such as endometrial cancer. AGT immunolabeling was highest in the stroma of the endometrium across the endometrial cycle and tended to be lower in the late-secretory phase compared to the mid-secretory phase. Immunolabeling of renin, Ang II, and AGTR1 are strongest within the proliferative and early secretory phases, coinciding with the highest *AGT* and *ATP6AP2* mRNA levels. The tendency for AGT immunolabeling to be lower in the late secretory phase further supports the idea that the renin–angiotensin system plays a regulatory role within the endometrial stroma, where it may drive stromal regeneration and proliferation. The potential role of the renin–angiotensin system in endometrial proliferation highlights the renin–angiotensin system as a clinical target in endometrial disorders and the importance of its inclusion in future investigations of proliferative endometrial diseases such as endometrial hyperplasia.

## Data Availability

The original contributions presented in the study are included in the article/**Supplementary Material**. Further inquiries can be directed to the corresponding author.
