# Supplementary material for: Characterization of elevated levels of endometrial renin–angiotensin system components suggest a role in endometrial repair
Source: Front Endocrinol (Lausanne). 2026 Apr 28;17:1817846. doi: 10.3389/fendo.2026.1817846 (PMC13160814; doi:10.3389/fendo.2026.1817846)
Supplement: Supplementary file 1 [file Table1.docx]

Supplementary Table 1

| **Official Gene Symbol** | **GenBank Accession Number** | **Primer Sequence (5'-3')** | **Concentration** |
| --- | --- | --- | --- |
| ***ACE*** | NM_000789 | Fw: CAGGTGGTGTGGAACGAGTATGC  Rv: TCTCTGTGGTGATGTTGGTGTTGTAGT | 200 nM |
| ***ACTB*** | NM_001101 | Fw: CGCGAGAAGATGACCCAGAT  Rv: GAGTCCATCACGATGCCAGT | 100 nM |
| ***AGT*** | NM_000029 | Fw: TCCAGCCTCACTATGCCTCT  Rv: GCTGGAAAGTGAGACCCTCC | 100 nM |
| ***ATP6AP2*** | NM_005765 | Fw: ACAATGAAGTTGACCTGCTCTTTCTTTCTG  Rv: CCTTGGCTAGATGCTTATGACGAGACA | 400 nM |
| ***REN*** | NM_000537 | Fw: AGTGGAGCCAACCCATGAAG  Rv: GCCATAGTACTGGGTGTCCAT | 200 nM |
| ***YWHAZ*** | NM_145690 | Fw: CCTGCATGAAGTCTGTAACTGAG  Rv: GACCTACGGGCTCCTACAACA | 100 nM |
| ***18sRNA*** | NR_145820 | Rv: TCTCTGTGGTGATGTTGGTGTTGTAGT  Rv: CCATCCAATCGGTAGTAGCG | 100 nM |

*ACE, Angiotensin Converting Enzyme 1; ACTB, β-Actin; AGT, Angiotensinogen; ATP6AP2, Prorenin Receptor; REN, Renin; YWHAZ, Tyrosine 3-Monooxygenase/Tryptophan 5-Monooxygenase Activation Protein Zeta; 18sRNA, 18s Ribosomal RNA.*
