# Supplementary material for: Characterization of elevated levels of endometrial renin–angiotensin system components suggest a role in endometrial repair
Source: Front Endocrinol (Lausanne). 2026 Apr 28;17:1817846. doi: 10.3389/fendo.2026.1817846 (PMC13160814; doi:10.3389/fendo.2026.1817846)
Supplement: Supplementary file 2 [file DataSheet1.docx]

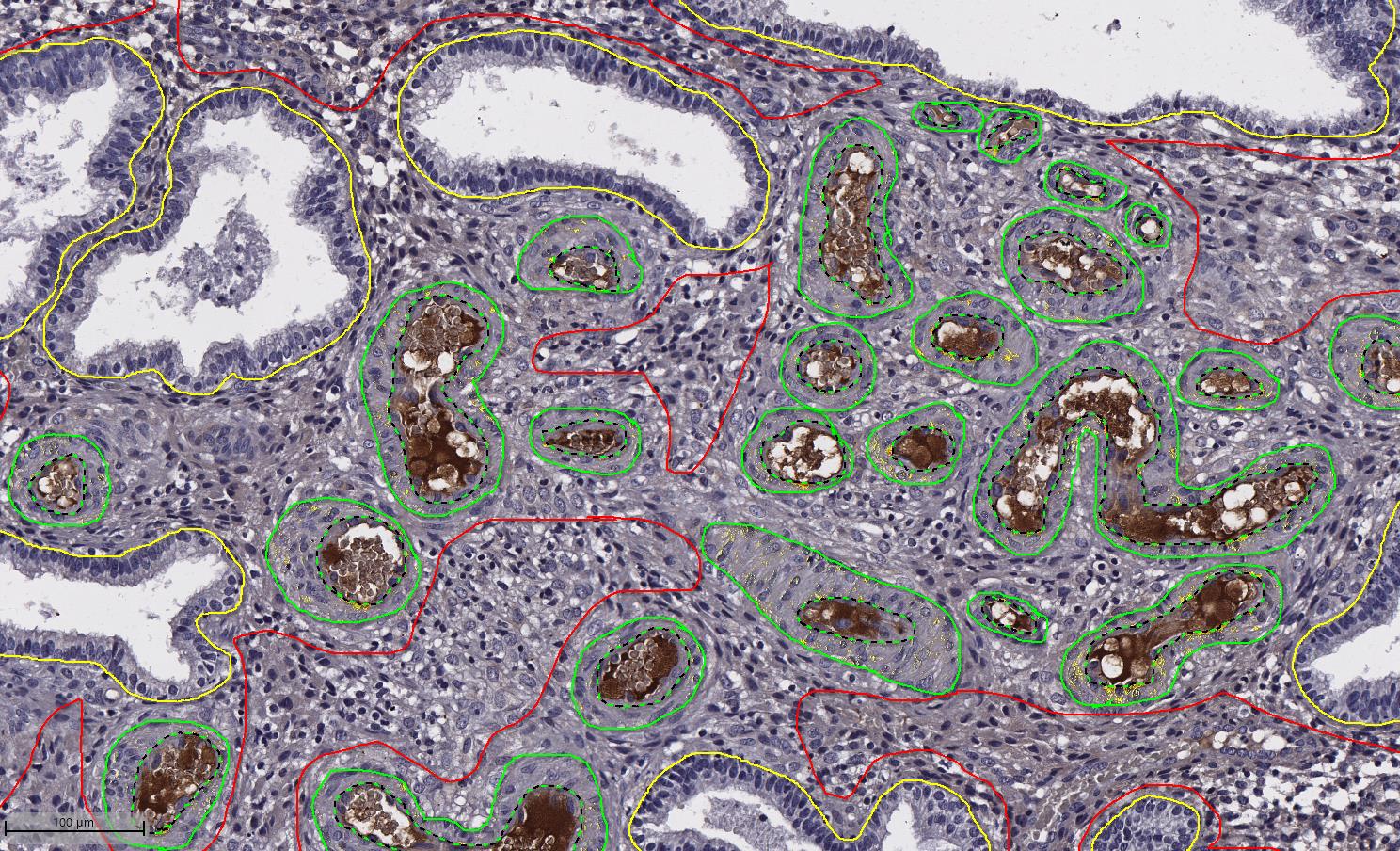


***Supplementary Figure 1.*** *Representative Image of Endometrial Tissue Segmentation.* HALO software was used to manually draw around each structure within the endometrium. The glandular epithelium was circled with yellow, the stroma with red, the endothelium was circled with green, and the luminal epithelium (not pictured) was circled with blue. For the endothelium, the scissor tool, represented by the green lines with black dashes, was used to exclude the staining of blood within the blood vessels.
